# Root canal contamination or exposure to lipopolysaccharide differentially modulate prostaglandin E _2_ and leukotriene B _4_ signaling in apical periodontitis

**DOI:** 10.1590/1678-7757-2019-0699

**Published:** 2020-05-11

**Authors:** Francisco Wanderley Garcia PAULA-SILVA, Fernanda Regina RIBEIRO-SANTOS, Igor Bassi Ferreira PETEAN, Maya Fernanda MANFRIN ARNEZ, Luciano Aparecido de ALMEIDA-JUNIOR, Fabrício Kitazono de CARVALHO, Léa Assed Bezerra da SILVA, Lúcia Helena FACCIOLI

**Affiliations:** 1 Departamento de Clínica Infantil Faculdade de Odontologia de Ribeirão Preto Universidade de São Paulo Ribeirão PretoSP Brasil Departamento de Clínica Infantil , Faculdade de Odontologia de Ribeirão Preto , Universidade de São Paulo , Ribeirão Preto , SP , Brasil .; 2 Departamento de Análises Clínicas, Toxicológicas e Bromatológicas Faculdade de Ciências Farmacêuticas de Ribeirão Preto Universidade de São Paulo Ribeirão PretoSP Brasil Laboratório de Inflamação e Imunologia das Parasitoses, Departamento de Análises Clínicas, Toxicológicas e Bromatológicas , Faculdade de Ciências Farmacêuticas de Ribeirão Preto , Universidade de São Paulo , Ribeirão Preto , SP , Brasil .; 3 Universidade de Pernambuco Arco VerdePE Brasil Universidade de Pernambuco , Arco Verde , PE , Brasil .

**Keywords:** Prostaglandin E _2_, Leukotriene B _4_, Apical periodontitis, Lipopolysaccharide, Root canal contamination

## Abstract

**Purpose:**

To evaluate the kinetics of apical periodontitis development *in vivo* , induced either by contamination of the root canals by microorganisms from the oral cavity or by inoculation of bacterial lipopolysaccharide (LPS) and the regulation of major enzymes and receptors involved in the arachidonic acid metabolism.

**Methodology:**

Apical periodontitis was induced in C57BL6 mice (n=96), by root canal exposure to oral cavity (n=48 teeth) or inoculation of LPS (10 µL of a suspension of 0.1 µg/µL) from *E. coli* into the root canals (n= 48 teeth). Healthy teeth were used as control (n=48 teeth). After 7, 14, 21 and 28 days the animals were euthanized and tissues removed for histopathological and qRT-PCR analyses. Histological analysis data were analyzed using two-way ANOVA followed by Sidak’s test, and qRT-PCR data using two-way ANOVA followed by Tukey’s test (α=0.05).

**Results:**

Contamination by microorganisms led to the development of apical periodontitis, characterized by the recruitment of inflammatory cells and bone tissue resorption, whereas inoculation of LPS induced inflammatory cells recruitment without bone resorption. Both stimuli induced mRNA expression for cyclooxygenase-2 and 5-lipoxygenase enzymes. Expression of prostaglandin E _2_ and leukotriene B _4_ cell surface receptors were more stimulated by LPS. Regarding nuclear peroxisome proliferator-activated receptors (PPAR), oral contamination induced the synthesis of mRNA for PPARδ, differently from inoculation of LPS, that induced PPARα and PPARγ expression.

**Conclusions:**

Contamination of the root canals by microorganisms from oral cavity induced the development of apical periodontitis differently than by inoculation with LPS, characterized by less bone loss than the first model. Regardless of the model used, it was found a local increase in the synthesis of mRNA for the enzymes 5-lipoxygenase and cyclooxygenase-2 of the arachidonic acid metabolism, as well as in the surface and nuclear receptors for the lipid mediators prostaglandin E2 and leukotriene B4.

## Introduction

The importance of microorganisms for the genesis of apical periodontitis was demonstrated in a classic study in which the dental pulp, when exposed to the oral environment in a germ-free mice, did not lead to bone loss; whereas in conventional laboratory animals lesions were detected after 15 days of exposure. ^1^ Until the 1970s, the isolation of microorganisms from root canals demonstrated a predominantly aerobic and anaerobic facultative composition. ^2^ Subsequently, it was observed that most of the microorganisms present in the infections of the root canal system of teeth with chronic periapical lesions were anaerobes, ^3 , 4^ particularly Gram-negative. ^2^ Gram-negative bacteria present bacterial lipopolysaccharide (LPS) or endotoxin as a component of the cellular wall, and contain both lipid components and polysaccharide moieties, with lipid A being considered the toxic portion of the molecule. ^5^ LPS is released during the occurrence of cellular stress, multiplication or bacterial death, stimulating a tissue immune-inflammatory reaction ^5 - 7^ and bone resorption. ^6 , 8 - 10^

Studies that perform the apical periodontitis induction procedure in their methodology show variations, either by the inoculation of a mixture of pathogens, ^11^ a single species such as *Fusobacterium nucleatum,* ^12^ LPS ^6^ or by the contamination of oral root canals by microorganisms from the oral cavity. ^13 , 14^ De Rossi, et al. ^11^ (2008) inducted the apical periodontitis by coronary opening, and inoculated a mixture of 4 pathogens ( *Porphyromonas gingivalis, Prevotella nigrescens, Actinomyces viscosus and Fusobacterium nucleatum subsp.polymorphum* ) into the root canals; later, the cavity was kept open to the oral environment. Unlike Wu, et al. ^12^ (2018) who performed the coronary opening and inoculated *Fusobacterium nucleatum* in a 2% carboxymethylcellulose vehicle, keeping the tooth cavity open for 21 days to the oral environment. In another study, carried out to analyze the effect of calcium hydroxide on bacterial endotoxins, the process of inoculating LPS into the root canal promoted extensive bone resorption. ^6^ Notwithstanding, another way of inducing periapical lesion is to perform a coronary opening, remove the pulp tissue and then leave the cavity open for contamination by microorganisms from the oral cavity. ^13 , 14^

During this response, biochemical mediators are released locally with the aim of stimulating cellular and humoral immune responses. Among these inflammatory mediators are eicosanoids, synthesized from the metabolism of arachidonic acid, produced by the action of phospholipase enzymes on the phospholipids present in cellular membrane. Cyclooxygenase (COX) and lipoxygenase (LO) enzymes cause structural modifications in arachidonic acid chain, leading to the synthesis of prostaglandins and thromboxanes or leukotrienes and lipoxins, respectively. ^15 - 18^

Prostaglandins are produced via COX-1 and COX-2 enzymes. ^19^ COX-1 is produced in physiological conditions while COX-2 is produced in response to several inflammatory stimuli, such as cytokines. This enzyme converts arachidonic acid to the intermediate isoform prostaglandin H _2_ (PGH _2_ ), which is converted to prostaglandin E _2_ (PGE _2_ ) by the prostaglandin E synthase microsomal enzymes 1 and 2 (mPGE-1 and mPGE-2). PGE _2_ acts on 4 different subtypes of membrane receptors (EP1, EP2, EP3 and EP4) coupled to G protein (Gαs, Gi and Gq) and, depending on the type of receptor stimulated, different cellular pathways are triggered. ^20^

In the presence of FLAP, a membrane-associated nuclear protein, the 5-LO enzyme is activated resulting in the oxidation of arachidonic acid to generate leukotriene B _4_ (LTB _4_ ). ^21^ LTB _4_ promotes chemotaxis of neutrophils, dendritic and T cells and increases vascular permeability. ^22^ LTB _4_ exerts its functions through cell surface receptors BLT1, which reveals high affinity for LTB _4_ , and BLT2. ^23^

Peroxisome proliferator-activated receptors (PPAR) are a family of nuclear receptors also activated by lipid mediators that play an important role as transcription factors in events such as inflammation, cell differentiation and lipid metabolism in macrophages and dendritic cells. ^24 , 25^ PPARα, PPARδ and PPARγ receptors are activated by ligands that modify their conformation, recruit transcription co-activators, and regulate gene transcription after binding to specific regulatory sites. ^26^

The hypothesis of this study was that oral contamination of the root canals would induce apical periodontitis similarly to LPS inoculation, and that enzymes and receptors involved in arachidonic acid would be involved in this process. Therefore, the aim of this research was to evaluate *in vivo* the kinetics development of apical periodontitis, induced by either contamination of the root canals by microorganisms from oral cavity or by inoculation of bacterial lipopolysaccharide (LPS) into the root canals. Because enzymes and receptors involved in the arachidonic acid metabolism are crucial in immune response, their expression in apical periodontitis was further investigated.

## Methodology

### Animals

C57BL/6 6-week-old male mice ( *Mus musculus* ; n=96) were used for experimentation after IRB approval (#12.1.60.53.8 and #13.1.266.53.6). Animals were anesthetized i.m. with ketamine hydrochloride (150 mg/kg; Ketamine 10%; National Pharmaceutical Chemistry Union Agener S/A, Embu-Guaçu, Brazil) and xylazine (7.5 mg/kg; Dopaser, Labs Calier S/A, Barcelona, Spain). Anesthesia was sustained throughout the experimental time.

### Operative procedures

Animals were placed on a surgical table with a device for mandibular retraction. The upper and lower first molars of each animal were used; on the right side it was induced apical periodontitis whereas the left side remained healthy. Occlusal root canal were accessed with 1011 spherical diamond burs (KG Sorensen Ind. com. Ltda., Barueri, Brazil), root canals were located with a #06 K-file (Les Fils d’ Auguste Maillefer S/A, Ballaigues, Switzerland) and the radicular pulp tissue was removed. Then, the animals were randomly assigned to experiment groups 1 and 2. In Group 1, root canals were left open to the oral environment for 7, 14, 21 and 28 days (n=12 teeth per group per period), as previously described. ^13^ In Group 2, 10 µL of a suspension of LPS (0.1 µg / µL) from *E. coli* 0127: B8 (L3129; Sigma-Aldrich Corp., St. Louis, USA) were inoculated into the root canals of each tooth using an automatic micropipette. The teeth were sealed with conventional glass ionomer cement (S.S. White Dental Articles Ltda, Rio de Janeiro, Brazil), mixed in accordance with the manufacturer’s instructions, and animals were followed-up for 7, 14, 21 and 28 days (n=12 teeth per group per period). In Group 3, healthy teeth were used as control (n=12 teeth per group per period). Animals were euthanized by i.m. anaesthetic overdose and tissues containing bone and tooth were collected for further analysis.

### Morphometric analysis of apical periodontitis size under light microscopy

An analysis was performed in hematoxylin and eosin-stained in all sections using the microscope at 10× magnification, in bright field. In each specimen, the size of the periapical lesion was delineated and the area was determined in μm ^2^ using the Software Zeiss AxioVision (Carl Zeiss AG Light Microscopy, Göttingen, Germany) in a Zeiss Axio Imager microscope (Carl Zeiss AG Light Microscopy) as previously described. ^13^ Lesion was delineated by excluding intact tooth and bone structures (periodontal ligament, cementum, and alveolar bone). Histopathological evaluation was performed by an experienced and blind examiner. Data were analyzed using two-way ANOVA followed by Sidak’s test (α=0.05).

### Quantitative reverse transcriptase-polymerase chain reaction (qRT-PCR)

To evaluate possible molecules involved in a periapical inflammatory response to the contamination or inoculation of LPS into the root canals, and considering the relevance of lipid mediators in inflammation, an investigation of two important pathways of arachidonic acid metabolism, COX-2 and 5-LO, was performed.

RNA was extracted from a pool of 3 teeth of the right and left upper first molars using the RNeasy Mini kit (RNeasy ^®^ Mini, Qiagen Inc., CA, USA) and samples were treated with DNAse I (RNase-Free DNase Set; Qiagen Inc.), according to manufacturer protocol. RNA integrity was analyzed using 1% agarose electrophoresis and quantity was estimated in NanoDrop 1000 (Thermo Fisher Scientific Inc., Wilmington, DE, USA) at 230, 260 and 280 ηm wavelenghts.

Complimentary DNA (cDNA) was synthesized from 1300 ng of total RNA using random primers (High Quality cDNA Reverse Transcriptase Kits, Applied Biosystems, Foster City, CA). Aliquots of 2 µl of the total cDNA were amplified by qRT-PCR using primers for *Ptgs2* (Mm00478374), *Ptger1* (Mm00443098), *Ptger2* (Mm00436051), *Ptger3* (Mm01316856), *Ptger4* (Mm00436053), *Alox5* (Mm01182747), *Alox5ap* (Mm00802100), *Ltb4r1* (Mm02619879), *Ltb4r2* (Mm01321172), *Ppara* (Mm00440939), *Ppard* (Mm00803184) and *Pparg* (Mm01184322) (TaqMan ^®^ Gene Expression Assay, Applied Biosystems) in an StepOne Plus equipment (Applied Biosystems). Gapdh (Mm99999915) was used as reference gene. qRT-PCR reactions were performed in duplicate, and amplification was done under the following conditions: denaturation at 95 °C for 2 min; followed by 40 cycles of 95°C for 1 s and 60°C for 20 s. Relative quantification was performed using the ΔΔCt Method. Data were analyzed using two-way ANOVA followed by Tukey’s test (α=0.05).

## Results

### Contamination or inoculation of LPS into the root canals, differentially induced inflammation and bone resorption

**Figure 1:**
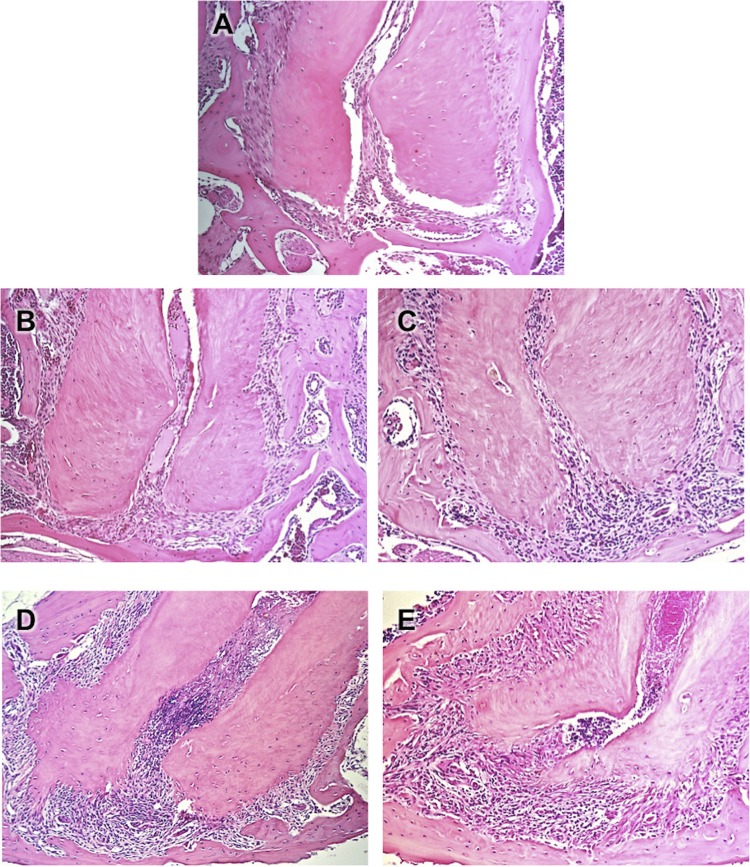
Photomicrographs representative of the periapical region of molars of healthy teeth from C57BL6 mice (A) and after contamination of the root canals by microorganisms from oral cavity at 7 (B), 14 (C), 21 (D) and 28 (E) days of the exposure. HE, 5x original magnification

**Figure 2:**
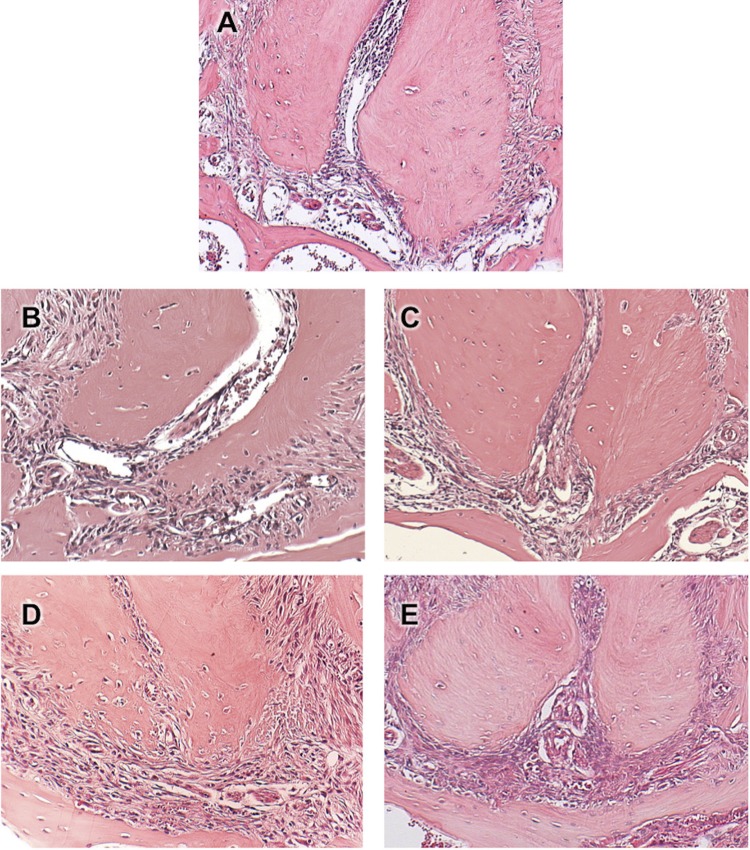
Photomicrographs representative of the periapical region of molars of healthy teeth from C57BL6 mice (A) and after inoculation of LPS (0.1 μg / μL) into the root canals at 7 (B),14 (C),21 (D) and 28 (E) days. HE, 10x original magnification

Contamination of root canals after coronary opening to the oral environment led to the development of apical periodontitis, initially characterized by the thickening of the apical periodontal ligament in the period of 7 days, the recruitment of neutrophils and macrophages at 14 days, the presence of bone resorption at 21 days, culminating in intense infiltration of inflammatory cells, edema and extensive periapical bone resorption at 28 days ( Figure 1 ). A different pattern was observed in root canals inoculated with LPS. Solely after 21 days a mild to moderate inflammatory infiltrate was detected, whereas bone resorption initiated at 28 days ( Figure 2 ). Healthy teeth were used as controls, in which an intact periodontal ligament could be observed, with fibers inserted in the cementum and in the alveolar bone.

**Figure 3:**
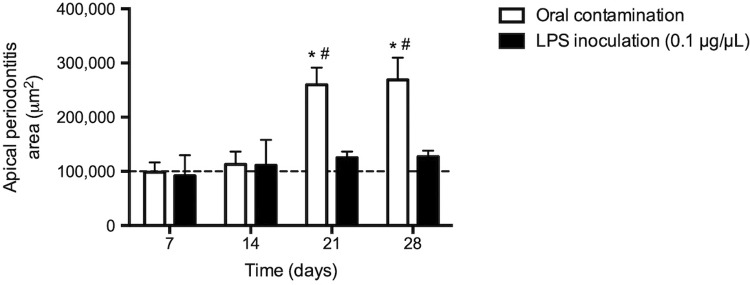
Measurement of apical periodontitis in μm 2 , evaluated at 7, 14, 21 and 28 days after contamination of root canals of molars of C57BL6 mice or after inoculation of LPS. * p<0.05 compared to the measurement of the periodontal ligament of healthy teeth (dashed line); # p<0.05 compared to inoculation of LPS

An increase in periapical space due to bone resorption was found in root canals exposed to oral contamination at 21 and 28 days after exposure, differently from root canals submitted to LPS inoculation or healthy teeth with a normal periodontal ligament and alveolar bone structure ( *p* <0.05, Figure 3 ).

Signalling signature of arachidonic acid metabolism enzymes and receptors modulated by contamination or inoculation of LPS into the root canals

**Figure 4:**
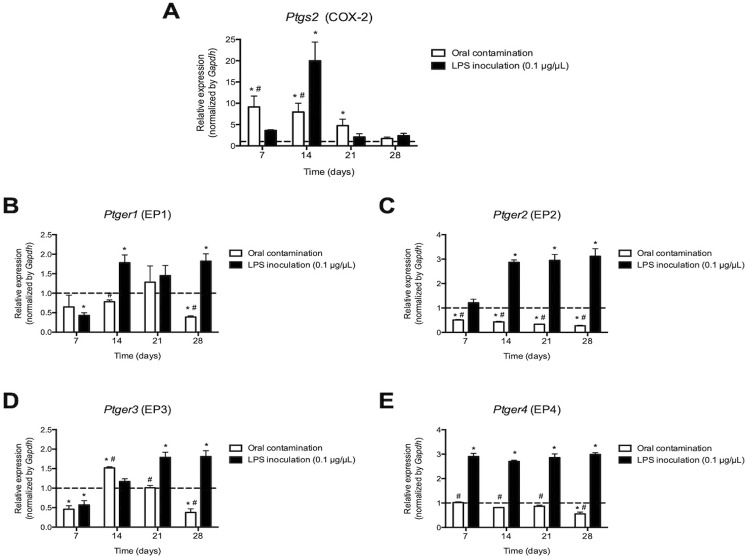
Expression of mRNA for cyclooxygenase-2 ( *Ptgs2* ) (A) and receptors for PGE2: EP1 ( *Ptger1* ) (B), EP2 ( *Ptger2* ) (C), EP3 ( *Ptger3* ) (D) and EP4 ( *Ptger4* ) (E), evaluated at 7, 14, 21 and 28 days after contamination of root canals of molars of C57BL6 mice or inoculation of LPS solution. * p<0.05 compared to baseline expression of the target genes in teeth without apical periodontitis (dashed line); # p <0.05 compared to the LPS group

Regarding the COX-2 pathway, it was observed that the *Ptgs2* gene, which encodes the COX-2 enzyme, showed low expression in the periapical tissues of healthy teeth. At 7 days after the contamination of the root canals, there was a significant increase in the expression of this gene, reaching the peak in this period and remaining higher up to 21 days ( *p* <0.05). Then, there was a reduction at 28 days, reaching values similar to the basal concentration observed in the teeth without apical periodontitis ( *p* >0.05). At 7 days after the inoculation of *E. coli* LPS into the root canals, there was a gradual increase in expression of the *Ptgs2* gene, reaching a peak expression at 14 days ( *p* <0.05). Then, there was an abrupt reduction in expression until reaching values similar to basal concentration ( *p* >0.05; Figure 4 ).

As for cell surface receptors for PGE _2_ , oral contamination did not modulate the expression of *Ptger1* , which encodes the EP1 receptor at 7, 14 and 21 days ( *p* >0.05), but inhibited expression at 28 days ( *p* <0.05). *Ptger2* , encoding the EP2 receptor, was inhibited throughout the experimental period ( *p* <0.05). On the other hand, oral contamination stimulated the expression of *Ptger3* , which encodes the EP3 receptor, at 14 days ( *p* <0.05), without altering its expression in other experimental periods ( *p* >0.05). *Ptger4* , which encodes EP4 receptor, was not modulated from 7 to 21 days ( *p* >0.05) but was inhibited at 28 days ( *p* <0.05). In contrast, inoculation of LPS in root canals inhibited expression of *Ptger1* at 7 days and stimulated expression at 21 and 28 days ( *p* <0.05). *Ptger2* and *Ptger4* were stimulated in all experimental periods ( *p* <0.05) and *Ptger3* was inhibited at 7 days and stimulated at 21 and 28 days ( *p* <0.05) ( Figure 4 ).

**Figure 5:**
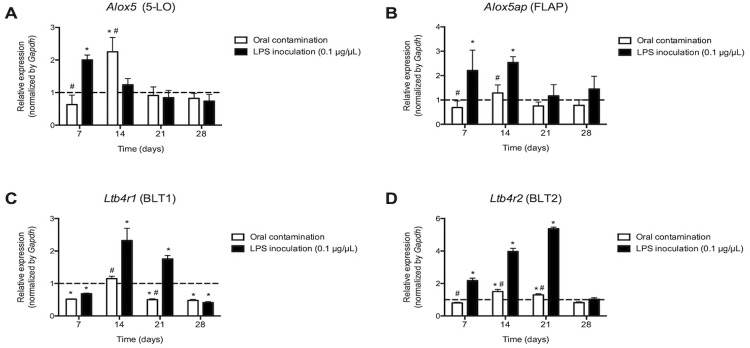
Expression of mRNA for the 5-lipoxygenase ( *Alox5* ) enzyme (A), for the 5-lipoxygenase activator protein ( *Alox5ap* ) (B) and receptors for LTB4: BLT1 ( *Ltb4r1* ) (C) and BLT2 ( *Ltb4r2* ) (D), evaluated at 7, 14, 21 and 28 days after contamination of root canals of molars of C57BL6 mice or after inoculation of a LPS solution. * p<0.05 compared to baseline expression of the target genes in teeth without apical periodontitis (dashed line); # p<0.05 compared to the LPS group

Regarding the 5-LO pathway, at 14 days after root canal contamination, there was an increase in expression of the *Alox5* gene, which encodes the 5-LO enzyme ( *p* <0.05), returning to baseline after this period ( *p* >0.05). The expression of *Alox5ap* gene encoding the 5-LO activating protein (FLAP) was not modulated by root canal contamination ( *p* <0.05). Differently, after the inoculation of LPS into the root canals, there was induction of *Alox5* at 7 days and *Alox5ap* at 7 and 14 days ( *p* <0.05). After these periods, there was a reduction in expression similar to that observed in teeth without apical periodontitis ( *p* >0.05) ( Figure 5 ).

As for cell surface receptors for LTB _4_ , oral contamination inhibited *Ltb4r1* , which encodes the BLT1 receptor, at 7, 21 and 28 days ( *p* <0.05), but stimulated expression of *Ltb4r2* , which encodes the BLT2 receptor, at 14 and 21 days after root canal exposure ( *p* <0.05). LPS inoculation, on the other hand, had an inhibitory effect (7 and 28 days) and stimulatory (14 and 21 days) on *Ltb4r1* and stimulatory on *Ltb4r2* (7, 14 and 21 days) ( *p* <0.05) ( Figure 5 ).

Fatty acids and derivatives, including a variety of eicosanoids (prostaglandins and leukotrienes), have been identified as ligands for nuclear PPAR receptors. Therefore, the expression of these receptors was investigated during kinetics development of apical periodontitis in order to identify receptors other than cell surface receptors (EP1-EP4 and BLT1-BLT2) that could be modulated by contamination or inoculation of LPS into the root canals.

**Figure 6:**
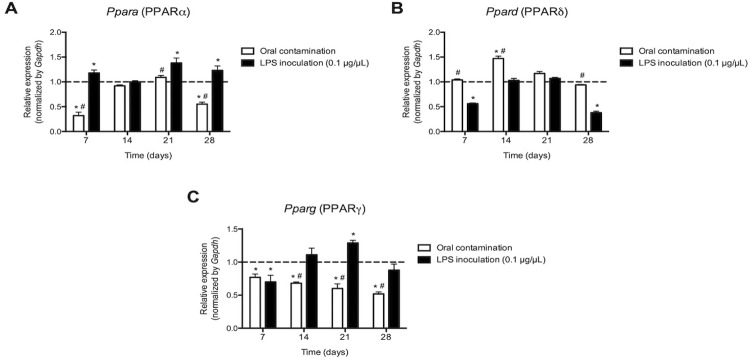
Expression of mRNA for the nuclear receptors PPARα ( *Ppara* ) (A), PPARδ ( *Ppard* ) (B) and PPARγ ( *Pparg* ) (C) for lipid mediators, evaluated at 7, 14, 21 and 28 days after root canals of molars of C57BL6 mice or after inoculation of a LPS solution. * p <0.05 compared to baseline expression of the target genes in teeth without apical periodontitis (dashed line); # p <0.05 compared to the LPS group

Oral contamination inhibited the expression of *Ppara* , which encodes the PPARα receptor at 7 and 28 days ( *p* <0.05), but did not alter expression at 14 and 21 days ( *p* >0.05). *Ppard* , which encodes the PPARδ receptor, was induced at 14 days ( *p* <0.05), without change in other periods ( *p* >0.05). *Pparg* , which encodes the PPARγ receptor, was inhibited in all evaluated periods ( *p* <0.05). LPS inoculation, on the other hand, induced *Ppara* at 7, 21 and 28 days ( *p* <0.05), inhibited *Ppard* at 7 and 28 days ( *p* <0.05) and induced *Pparg* at 21 days ( *p* <0.05). In other periods, there was no modulation of nuclear receptors by LPS ( *p* >0.05) ( Figure 6 ).

## Discussion

Our results showed that oral contamination leads to the development of apical periodontitis, characterized by the recruitment of inflammatory cells, tissue destruction and bone resorption, while the inoculation of LPS, in the concentration used, induces cell recruitment without periapical bone loss. Previous studies reported that the composition of microorganisms of root canal infections includes Gram-negative bacteria, which releases an endotoxin called bacterial lipopolysaccharide LPS, that has lipid A component that is considered its toxic portion. ^2 , 5 - 10^ Thus, we reject the initial hypothesis that induction of apical periodontitis is similar when using inoculation with LPS or contamination of the root canal by oral cavity microorganisms.

Our study was carried out to induce apical periodontitis by coronary access, insertion of a file into the root canals and pulp extirpation, according to previous studies. ^13 , 14 , 27^ However, to achieve the induction of the apical periodontitis, other studies use either coronary opening and disorganization of the coronary pulp, ^28 , 29^ a pool of several microorganisms into the root canals, ^13^ a combination of a pool of bacteria and oral contamination, ^11^ a single specific bacteria such as *F. nucleatum* ^12^ or bacterial products such as LPS. ^6 , 8^

In this study, contamination or inoculation of LPS into the root canals stimulated the expression of genes encoding enzymes and receptors involved in the metabolism of arachidonic acid. The presence of prostaglandins and arachidonic acid metabolism enzymes in the development of apical periodontitis was previously demonstrated after exposure of the pulp tissue to the oral environment or inoculation of LPS into the root canals in animal experimental models ^30 - 32^ or in humans. ^33 , 34^ Macrophages were identified as the main cellular source responsible for the synthesis of prostaglandins. ^34^ Furthermore, active osteoclasts were observed along the alveolar bone surface, suggesting that prostaglandins produced by macrophages could modulate the activity of osteoclasts and contribute to the resorptive activity in apical periodontitis. ^34^ Similarly, after induction of apical periodontitis in rat teeth through oral contamination for 5, 10, 15 and 20 days, it was observed that macrophages and osteoblasts showed positive staining for COX-2 and significantly increasing COX-2 expression from 5 to 20 days. ^35^ These results differ from ours, since gene expression was higher in the initial periods of development of apical periodontitis, regardless of whether the induction was performed by means of LPS inoculation or oral contamination.

Most of the actions mediated by PGE _2_ are performed after activation of one of its surface receptors called EP1, EP2, EP3 and EP4. ^36^ cDNA cloning studies have shown that all these receptors have seven characteristic transmembrane domains of G protein coupled receptors, although each is encoded by a specific gene (i.e., *Ptger1, Ptger2, Ptger3* and *Ptger4* ), and promote cellular responses by the activation of different types of G protein (inhibitory or stimulatory). In this study, an increase in the expression of all the receptors for PGE _2_ was observed, after the inoculation of LPS into the root canals, when compared to the control group. Contamination of root canals inhibited or did not modulate the expression of EP1-EP4 receptors, except *Ptger3* that was induced at 14 days.

Metabolites derived from 5-LO pathway may also represent important mediators of cell recruitment, tissue inflammation and bone resorption. ^13^ Specifically, two products of the 5-LO pathway (leukotrienes B _4_ and C _4_ ) were identified in human periapical lesions ^37^ and higher expression of LTB _4_ was positively correlated clinically with the presence of symptomatic pain, and histologically with the presence of polymorphonucleated inflammatory cells. ^37^ In the present study, after the inoculation of LPS or the contamination of the root canals, it was observed an increase in expression of *Alox5* gene, encoding for the 5-LO enzyme, and a decrease in mRNA expression for this enzyme at 21 and 28 days. Regarding the receptors for LTB _4_ , buccal contamination induced the gene expression of the PPARδ nuclear receptor and the BLT2 surface receptor, but inhibited the gene expression of BLT1, PPARα and PPARγ. Given that pattern recognition receptors (PRRs) mediate the recognition of the microorganisms by the cells of the innate immune system, generating secondary signals that transduce the signals from the cell surface, we hypothesize that the products of the 5-LO pathway can act both in the extracellular medium and directly within the cell to trigger host response. In a model of ligand-induced periodontal disease, treatment with a PPARδ nuclear receptor agonist (GW0742) produced less inflammatory cytokines, inducible nitric oxide synthase (iNOS) enzyme, apopotosis, and tissue damage, indicating that the receptor mediates anti-inflammatory events. ^38^

In previous studies, the inoculation of LPS in a model of periodontal disease ^31^ or induction of apical periodontitis ^6 , 39^ resulted in increased osteoclastic function and bone resorption, differently from that observed in the present study. This divergence can be attributed to two important factors. First, the concentration of LPS used in this study (0.1 μg / μL), ^32^ which resulted in a mass of approximately 1 μg LPS per tooth, compared to 25 μg used in a previous study. ^39^ Another important factor is the time required for induction of periapical bone resorption. The present study was performed in 28 days, which may not have been enough to detect the effects of the used concentration of LPS on bone resorption. Nonetheless, we used histology to find a difference between groups, and we speculate that this difference would be even greater using computed tomography to investigate the area and volume of the periapical lesion, as previously demonstrated. ^28 , 40^

## Conclusion

Contamination of the root canals by microorganisms from oral cavity induced the development of apical periodontitis differently than by inoculation with LPS, characterized by less bone loss than the first model. Regardless of the model used, it was found a local increase in the synthesis of mRNA for the enzymes 5-lipoxygenase and cyclooxygenase-2 of the arachidonic acid metabolism, as well as the surface and nuclear receptors for the lipid mediators prostaglandin E2 and leukotriene B4.
